# Supplementary material for: Bioactivity Profiling of Daedaleopsis confragosa (Bolton) J. Schröt. 1888: Implications for Its Possible Application in Enhancing Women’s Reproductive Health
Source: Pharmaceuticals (Basel). 2024 May 8;17(5):600. doi: 10.3390/ph17050600 (PMC11123820; doi:10.3390/ph17050600)
Supplement: Supplementary file 1 [file pharmaceuticals-17-00600-s001.zip › pharmaceuticals-2989753-supplementary.pdf]

Table S1. Extraction yield of *D. confragosa* analyzed EtOH extracts.

Sample	Yield of Extracts	Extraction
	**g/10 g d.w.**	**% (*w/w*)**
D. confragosa (DC)	0.31 ± 0.05	3.09
D. confragosa HD (DCHD)	0.006 ± 0.05	0.63

HD - this extract is prepared using the hydrodistillation technique. The data is presented as the mean ± standard deviation of triplicate measurements.

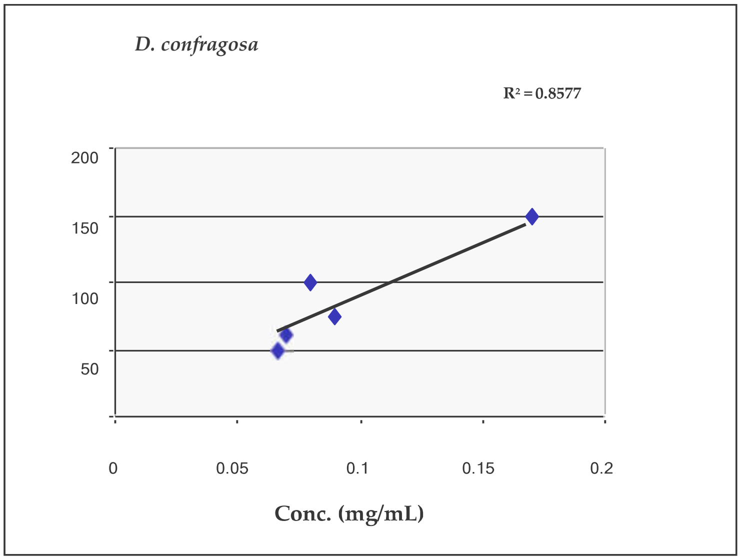

Figure S1. Dependence of the reciprocal value of the hemolysis rate $1/t_{0.5}$ (min) on the extract concentration (mg/mL).
